# Perturbation of gene expression of the chromatin remodeling pathway in premature newborns at risk for bronchopulmonary dysplasia

**DOI:** 10.1186/gb-2007-8-10-r210

**Published:** 2007-10-04

**Authors:** Jennifer Cohen, Linda J Van Marter, Yao Sun, Elizabeth Allred, Alan Leviton, Isaac S Kohane

**Affiliations:** 1 Children's Hospital, Boston, Division of Newborn Medicine, Boston, Longwood Avenue, Boston, MA 02115, USA; 2 Brigham and Women's Hospital Division of Newborn Medicine, Boston, Longwood Avenue, Boston, MA 02115, USA; 3 Kaiser Permanente Santa Clara, Santa Clara, Lawrence Expressway, Santa Clara, CA 95051, USA; 4 Children's Hospital Boston, Neuroepidemiology Unit, Boston, Longwood Avenue, Boston, MA 02115, USA; 5 Harvard School of Public Health, Boston, Longwood Avenue, Boston, MA 02115, USA; 6 Children's Hospital Informatics Program at the Harvard-MIT Division of Health Sciences and Technology, Boston, Longwood Avenue, Boston, MA 02115, USA; 7 Harvard-Partner's Center for Genetics and Genomics, Boston, Longwood Avenue, Boston, MA 02115, USA

## Abstract

**Background:**

One-third to one-half of all infants born before the 28th week of gestation develop bronchopulmonary dysplasia (BPD). Inflammatory regulators appear to be involved in the pathogenesis of BPD, possibly beginning in fetal life. To evaluate the feasibility of using expression profiling in umbilical cord tissue to discover molecular signatures for developmental staging and for determining risk of BPD, we conducted a cross-sectional study of infants born at less than 28 weeks of gestation (*n *= 54). Sections of umbilical cord were obtained at birth from 20 infants who later developed BPD and from 34 of their peers who did not develop BPD.

**Results:**

Umbilical cord expression profiles at birth exhibited systematic differences in bioenergetic pathways with respect to gestational age. Infants who subsequently developed BPD had distinct signatures involving chromatin remodeling and histone acetylation pathways, which have previously been implicated in several adult onset lung diseases. These findings are consistent with prior work on inflammatory processes and bioenergetics in prematurity.

**Conclusion:**

This study of gene expression of the newborn umbilical cord implicates the chromatin remodeling pathways in those premature infants who subsequently develop BPD. Larger sample sizes will be required to generate prognostic markers from umbilical cord profiles.

## Abstract 2

The expression profiles of umbilical cords from premature newborns reveal distinct patterns, including changes in the expression of chromatin remodeling factors, associated with the development of bronchopulmonary dysplasia.

## Background

The lung disorder bronchopulmonary dysplasia (BPD) occurs in 20% to 40% of infants born at under 1,000 g and before 28 completed weeks of gestation [1-4] and it is the second leading cause of death among infants born within this gestational age [5]. Previously identified prenatal factors that are associated with the development of BPD include surfactant deficiency and maternal infection, including chorioamnionitis [6]. Among the multiple postnatal environmental factors and neonatal conditions that have been linked to the development of BPD are supplemental oxygen exposure, barotrauma from ventilation, the presence of patent ductus arteriosus, and neonatal infection.

Little attention has been directed to the genetics of BPD risk or the physiologic pathways, measurable at the time of delivery, that predispose preterm infants to BPD. Inflammatory mediators, including cytokines and growth factors, appear to be involved in the development of BPD [7], and their activity can be upregulated by inflammatory processes that might begin prenatally and affect the fetus via the placenta [6,8]. For example, chorioamnionitis and funisitis are accompanied by alterations in the expression of inflammatory mediators [9].

The placenta and umbilical cord tissue appear both to reflect and influence the intrauterine environment. What remains unclear is the extent to which biologic markers obtained from the umbilical cord might have clinical and developmental correlates in the fetus in both the prenatal and perinatal periods. Given the multiple tissue types in the umbilical cord that are also present in the placenta [10-12], we hypothesize that the umbilical cord genomic signatures may relate to the overall physiologic state of the maternal-infant unit. In this study we have explored this hypothesis by examining the relationships between human gene expression in the umbilical cord at the time of birth and subsequent BPD, defined as the requirement for supplemental oxygen at 36 weeks postmenstrual age. This also allowed us to identify characteristics of the preterm infant at risk for developing BPD.

## Results

Of the 72 umbilical cord samples collected for this study, 54 contained at least the minimum of 7 μg RNA required for hybridization (13 did not) and were from infants who survived to 36 weeks postmenstrual age (5 died), and therefore at risk for BPD. All expression data are available in the National Center for Biotechnology Information's Gene Expression Omnibus repository (GSE8586).

The infants included in this study were slightly more mature than those in the Extremely Low Gestational Age Newborn (ELGAN) study [13] as a whole (Table 1). This may explain why they had a higher survival rate and lower incidences of BPD and retinopathy of prematurity.

**Table 1:** Clinical outcomes of the ELGAN (*n *= 1508) study population as compared with the study sample (*n *= 54)

Outcome	ELGAN	Current cohort	*P*
Mortality	21%	11%	0.553
BPD	50%	37%	0.095
NEC (definite disease)	7%	7%	0.459
ROP	62%	48%	0.041
IVH	25%	23%	0.749
GA < 27 weeks	71%	52%	< 0.01

BPD, bronchopulmonary dysplasia; ELGAN, Extremely Low Gestational Age Newborn; NEC, Necrotizing Entercolitis (a serious infection of the bowel); IVH, Intraventricular Hemorrhage (bleeding in the brain); ROP, Retinopathy of Prematurity (a disease that affects immature vasculature in the eyes of premature babies causing significantly decreased vision); GA, Gestational Age.

Infants with and without BPD exhibited minimal differences in maternal characteristics, including cause of delivery, antenatal steroid exposure, race, or histopathologic evidence of chorioamnionitis (Table 2). Infants who developed BPD were of lower birth weight and less mature gestational age, required more days of supplemental oxygen and ventilation, and had higher rates of retinopathy of prematurity. They did not differ in sex ratio or rates of patent ductus arteriosus, and had a modestly higher rate of sepsis (Table 3).

**Table 2:** Baseline characteristics of mothers and placentas related to infant outcomes in terms of BPD

Characteristic	No BPD (*n *[%])	BPD (*n *[%])	*P*
Preterm labor	20 (58.8)	8 (40)	0.146
Pregnancy-induced hypertension	2 (5.9)	4 (20)	0.127
Cesarean section	26 (76.5)	16 (80)	0.522
Antenatal glucocorticoids	21 (61.8)	14 (70)	0.379
Nonwhite race	11 (32.4)	9 (45)	0.26
Chorioamnionitis			
Neutrophils in plate	9 (26.5)	7 (38.9)	0.27
Neutrophils in cord	13 (40.6)	5 (33.3)	0.441

BPD, bronchopulmonary dysplasia.

**Table 3:** Characteristics of infants in relation to the primary outcome BPD

Characteristic	No BPD	BPD	*P*
Birth weight (g; median [range])	975 (532 to 1,360)	695 (460 to 1,080)	< 0.005
Gestational age < 27 weeks (*n *[%])	13 (38.2)	15 (75)	0.009
Male (*n *[%])	19 (55.9)	12 (60)	0.497
Sepsis (suspected or confirmed; *n *[%])	2 (5.9)	3 (15.8)	0.239
PDA (suspected or definite; *n *[%])	24 (72.7)	14 (73.7)	0.603
ROP (*n *[%])	13 (38.2)	13 (68.4)	0.03
Supplemental oxygen (days; median [range])	31.5 (2 to 88)	68 (37 to 91)	< 0.005
Mechanical ventilation (days; median [range])	3 (0 to 38)	37 (1 to 77)	< 0.005

BPD, bronchopulmonary dysplasia; ROP(Retinopathy of Prematurity, a disease that affects immature vasculature in the eyes of premature babies leading to significantly decreased vision). PDA (Patent Ductus Arteriosus, a persistent connection between the aorta and the pulmonary artery).

**Table 4:** Differentially expressed gene sets for gestational age under 27 weeks versus 27 to 28 weeks

Gene set/pathway	NT_k _*q *value	NE_k _*q *value
Coenzyme metabolism (GO:0006731)	0.0	0.0
Mitochondrial inner membrane (GO:0005740)	0.0	0.0
Ribonucleotide biosynthesis processing (GO:0009260)	0.0	0.0
Mitochondrial matrix (GO:0005759)	0.0	0.08
Citrate cycle TCA cycle (GO:0006099)/Oxidative phosphorylation (KEGG)	0.0	0.1

Shown here are the pathways (with redundant pathways removed) that were ranked at the top of the list of those differentially expressed by two measures of false discovery: the NT_k _*q *value and the NE_k _*q *value. The NT_k _*q *value corresponds to the degree to which genes within a set/pathway are more predictive of the phenotype than the genes outside that set, and the NE_k _*q *value corresponds to the degree to which genes within that set are predictive of the phenotype. Only those pathways with an NT_k _*q *value below 10^-4 ^and a NE_k _*q *value below 1 are shown above. The relatively high NE_k _*q *value with respect to NT_k _*q *value values shows that these gene sets are not as significantly predictive of outcome, because they demonstrate significant differential expression with respect to the other gene sets. This illustrates the limitations of the sample size and nature of the current data set. Each pathway is annotated by ontology source (Gene Ontology [GO] or Kyoto Encyclopedia of Genes and Genomes [KEGG]). TCA, tricarboxylic acid.

**Figure 1:**
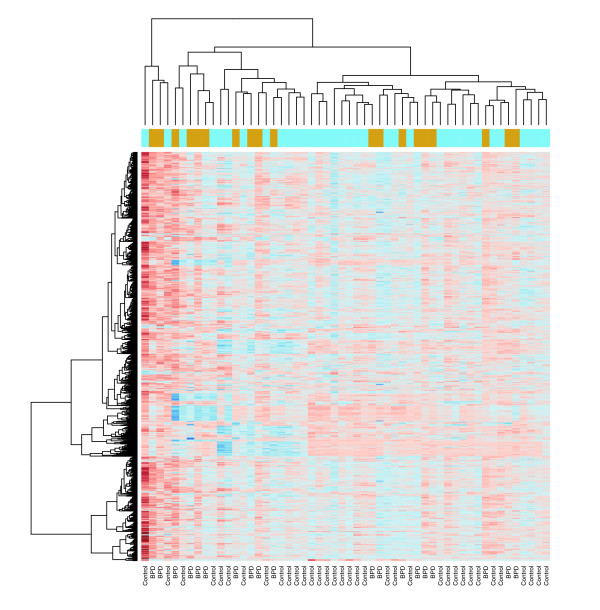
Unsupervised clustering based on Euclidean distance in expression between samples. Each row corresponds to a gene and each column (labeled at the bottom) corresponds to an infant who subsequently developed bronchopulmonary dysplasia (BPD) or a control infant.

**Figure 2:**
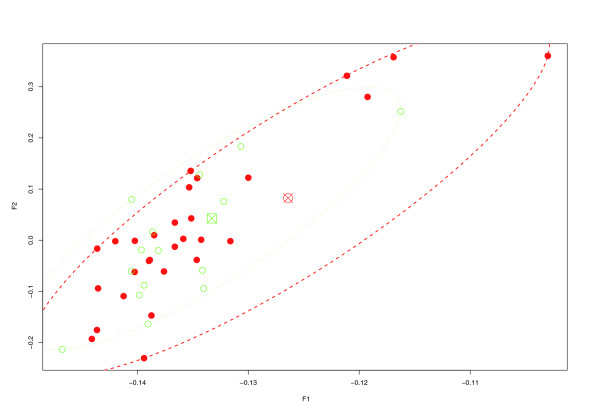
Principal component analysis of the samples at birth. The closed circles correspond to infants who did not develop bronchopulmonary dysplasia (BPD) and the open circles to those who did develop BPD. The centroid of the ellipsoid hull for each subgroup is illustrated with a symbol over-struck with an X (circle for controls and square for BPD). This plot illustrates the limited separation of these samples from the transcriptome-wide perspective. *P *> 0.05 for *t*-test in each of the principal components.

Unsupervised learning revealed inhomogeneous clustering of the sample by gestational age (27 to 28 weeks versus < 27 weeks; data not shown) and by presence of BPD (BPD versus control; Figure 1). Similarly, principal components analysis of the 54 samples across the 54,675 measured transcripts revealed partial overlap of the samples across the two groups (Figure 2). Single gene differential gene expression analysis revealed no genes with a *q *value (a measure of false discovery rate [FDR] [14]) below 0.05 or an uncorrected *P *value below 10^-6^. The high FDR confirmed that the overlap between the two classes of infants was high; therefore, we explored systematic differences in pathways rather than individual genes, as others have done with similarly subtle differences across human samples [15].

The gene pathways most differentially expressed by gestational age category were those related to oxidative phosphorylation, mitochondrial energy metabolism, and DNA repair (Table 4). None of the genes within these pathways by themselves were significantly differentially expressed, but the *q *value for the entire set of genes within each pathway (of the more than 600 sets evaluated) was below 0.0001, as calculated using the sigPathway package [16].

**Figure 3:**
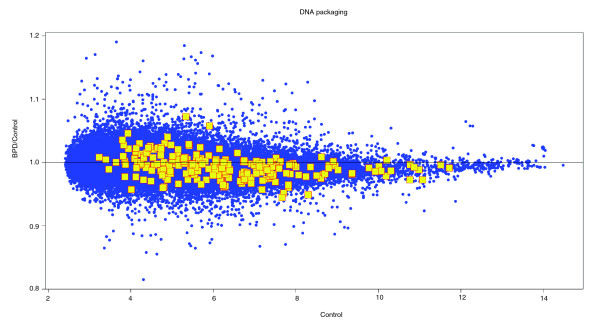
Relative expression of DNA packaging gene set relative to the overall transcriptome. The ratio of expression level of each gene in samples of infants that went on to develop bronchopulmonary dysplasia (BPD) against those who did not is plotted against the expression value in the infants without BPD. Overall, the entire transcriptome (small closed circles) is distributed around the horizontal 1.0 line (equal in BPD and control infants). However, the 229 members of the DNA packaging gene set (large squares) are significantly biased below the 1.0 line (NT_k _*q *value = 0). This figure illustrates that, individually, none of the DNA packaging gene set fell significantly outside the range of variation of the entire transcriptome.

**Figure 4:**
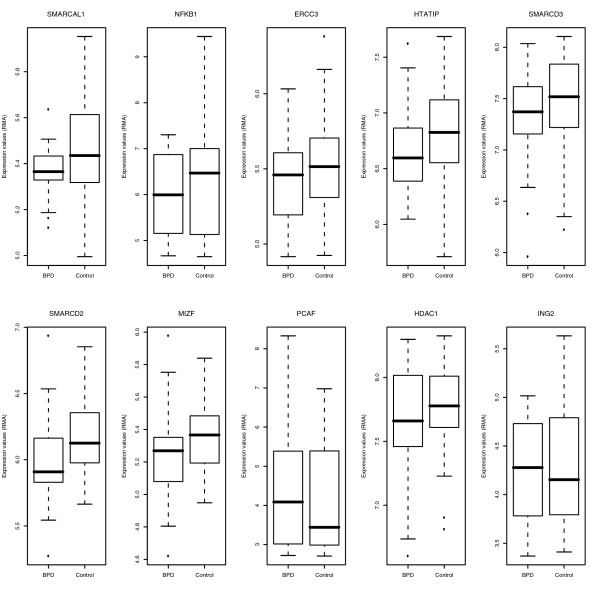
Box-plots of differential expression of the ten most differentially expressed genes in SWI/SNF chromatin remodeling pathway. Individually, none of these genes reached significance for differential expression, although the pathway was highly significantly enriched.

When the samples were evaluated comparing infants with BPD versus those without BPD, the most differentially expressed gene pathways included the aforementioned bioenergetic pathways, as well as histone acetyltransferase binding activity and chromatin remodeling pathways (Table 5). Although the individual genes within these pathways were not significantly differentially expressed, the expression of the entire 'chromatin packaging' pathway relative to the overall transcriptome was highly significantly differentially expressed (Figure 3). The ten most differentially expressed genes of those in chromatin remodeling pathways are shown in Figure 4 to illustrate how this finding is only a trend at the individual gene level.

**Table 5:** Differentially expressed pathways in infants with BPD versus infants without BPD

Gene set/pathway	NT_k _*q *value	NE_k _*q *value
Regulation of cell growth (GO:0001558)	0.0	0.0
Nuclear pore complex (GO:0046930)	0.0	0.03
Negative regulation of cellular metabolism (GO:0031324)	0.0	0.04
Chromatin remodeling (GO:0016568)	0.0	0.055
PI3K-AKT signaling pathway (humanpaths)	0.0	0.1
Histone acetyltransferase binding (GO:0035035)	0.0	0.2
cAMP/Ca^2+ ^signaling (humanpaths)	0.0	0.3
Spliceosome complex (GO:0005681)	0.0	0.9

Shown here are the pathways (with redundant pathways removed as well as those that overlapped with Table 4) that were ranked at the top of the list of those differentially expressed by two measures of false discovery: the NT_k _*q *value and the NE_k _*q *value. The NT_k _*q *value corresponds to the degree to which genes within a set/pathway are more predictive of the phenotype than the genes outside that set, and the NE_k _*q *value corresponds to the degree to which genes within that set are predictive of the phenotype. Only those pathways with an NT_k _*q *value below 10^-4 ^and a NE_k _*q *value below 1 are shown above. Each pathway is annotated by ontology source (Gene Ontology [GO] or humanpaths). BPD, bronchopulmonary dysplasia; PI3K, phosphoinositide-3 kinase.

## Discussion

Microarray profiling has been used to classify and predict human disease, but this technique has not yet been applied widely to the investigation of diseases of the premature newborn infant. Expression analysis has proved informative in murine models of fetal and postnatal lung injury [17]. Neonatal lung samples, however, are not routinely available. Umbilical cords are routinely available, and their availability has allowed us to explore expression profiles at birth with respect to gestational age and subsequent development of BPD. This has provided a rare opportunity to examine the influence of fetal physiology on postnatal health and development using the multiple tissues in umbilical cords as a proxy for a wide variety of tissues in the maternal-fetal unit.

Gene expression signatures alone provide imperfect clustering of different gestational age groups in the unsupervised analyses. For this reason, as expected, the single gene differential expression measures were fraught with high false discovery rates. However, the supervised analysis at the level of entire pathways [15] revealed highly significant distinctions for both gestational age and for the subsequent development of BPD.

Several biologic pathways have emerged from this investigation, characterizing different early physiologic states: bioenergetics (Krebs cycle, mitochondrial function, and oxidative phosphorylation), transporter activity, DNA synthesis and repair, and chromatin remodeling. However, the latter pathway was consistently present at a frequency greater than expected by chance alone (it was enriched) in the comparisons of all infants with BPD compared with control infants. These findings are consistent with the earlier referenced single gene investigations of prematurity implicating inflammatory and bioenergetic processes [6-9,18].

### Chromatin remodeling

The chromatin remodeling apparatus is involved in the inflammatory pathways in adult lung disease [19]. Indeed, glucocorticoids (one of the mainstays of therapy for pulmonary diseases) reverses histone acetylation of activated inflammatory genes by binding liganded glucocorticoid receptors to coactivators, by recruiting histone deacetylase-2 to the activated transcription complex [19-21]. However, to our knowledge, this is the first documentation in human preterm neonates of the relative activity of the histone acetylation/chromatin remodeling pathway at birth in individuals who subsequently develop BPD. This intersection with the pathophysiology of chronic obstructive pulmonary disease, although very limited, does raise the question of whether a subset of infants with BPD and adults with chronic obstructive pulmonary disease share a common vulnerability that is exposed by different stressors. Such commonality might have practical import for prevention and treatment [21].

In our study, the proportions of infants exposed to antenatal glucocorticoids were similar in infants who did and those who did not develop BPD. Thus, differential administration of corticosteroids did not distort the relationship between gene expression and development of BPD.

### Bioenergetic pathways

Differentially expressed energy pathways seen in younger premature infants when compared with older premature infants suggest that the timing of delivery may have a global effect on energy metabolism. Although human data are lacking, preterm rats tend to have diminished mitochondrial content and function compared with term born rats [22]. The sets of genes characteristic of oxidative phosphorylation and other bioenergetics were expressed at lower levels in BPD infants than in control infants and are also developmentally regulated, with lower levels of expression in the least mature. Consequently, in this small sample we could not separate a gestational age effect from a BPD propensity.

In summary, this study of RNA expression profiles in umbilical cord tissue demonstrates the potential of this technology in investigations of perinatal and postnatal disorders. Because chromatin remodeling pathways appeared to be differentially regulated in umbilical cord tissues of the subsequently BPD-affected neonates, therapeutic modalities that are being explored for treatment of adult pulmonary diseases with similar molecular pathophysiology (for example, steroids with fewer side effects such as RU24858 and RU40066 [21] and antioxidants [23,24]) may hold considerable new promise for those infants who are at risk for BPD. The analytic methodologies used here might also be sufficiently robust to identify individuals who are at risk. Although entire pathways were significantly differentially regulated between BPD and control infants, multigenic predictors based on these pathways did not exhibit strong performance, and identification of more predictive biomarkers will require larger premature neonate sample sizes

## Materials and methods

The study population consisted of a subset of preterm infants born at one of three centers (Brigham and Women's Hospital, Beth Israel Deaconess Medical Center, and Wake-Forest Medical Center) between 1 April 2004 and 31 August 2004. The included infants were at 23 to 28 weeks gestational age, for whom parental consent to participate in the ELGAN study had been granted and from whom supplemental umbilical cord specimens were available. Each hospital's institutional review board approved the study. Mothers of potential participants signed a consent form when they were admitted to the high-risk obstetric service and before the time of delivery. Enrollment of infants took place at the time of birth.

Specimens were collected as part of the ELGAN study. A study nurse collected umbilical cord tissue samples at the time of delivery. Within 1 hour of collection, samples were flash frozen to -70°C.

Total RNA was isolated from umbilical cord tissue homogenates. Tissue samples were homogenized in 1 ml TRIZOL reagent using a power homogenizer. Chloroform (0.2 ml) was then added. Samples were centrifuged at no more than 12,000 *g *for 15 min at 2 to 8°C. RNA was precipitated from the aqueous phase by mixing with 0.5 ml isopropyl alcohol and centrifuged at no more than 12,000 *g *for 10 min at 2 to 8°C. RNA pellet was washed with 1 ml of 75% ethanol. RNA was dissolved in 100 μl RNase-free water. The solution was re-suspended in 100 μl water and incubated at 37°C for 5 min. Qiagen RNeasy Mini Kit (Qiagen Inc. Valencia, CA, USA) along with 350 μl Buffer RLT (with B-mercaptethanol) was added to 100 μl dissolved RNA. Then, 250 μl ethanol (96% to 100%) was added to the diluted RNA. The sample (700 μl) was applied to an RNeasy mini column placed in a 2 ml collection tube, and then centrifuged at ≥ 8,000 *g *(≥ 10,000 rpm). buffer RPE (500 μl) was pipetted onto the RNeasy column and then centrifuged at ≥ 8,000 *g *(≥ 10,000 rpm). Northern blot analysis was performed on each sample to confirm quality. AffymetrixÆ U133 chips (Affymetric Inc., Santa Clara, CA, USA) were used for hybridization.

Demographic and clinical information was gathered from patient charts, ELGAN clinical data collection sheets, and maternal interviews. The primary outcome of BPD was defined as a persistent oxygen requirement at 36 weeks postmenstrual age.

### Computational analysis

Samples were evaluated by outcome of BPD and by gestational age. Single gene analysis was performed using the multtest and qvalue packages from Bioconductor [25] to identify those genes that were differentially expressed by the *t *test and ranked by FDR.

Gene set enrichment analysis was conducted using the Sigpathway package of Tian and coworkers [16] using 1,000 permutations for each analysis. Only the top ranked pathways with an NT_k _*q *value below 10^-4 ^and an NE_k _*q *value below 1.0 were included in the results. The NT_k _*q *value corresponds to the degree to which genes within a set/pathway are more predictive of the phenotype than the genes outside that set, and the NE_k _*q *value corresponds to the degree that genes within that set are predictive of the phenotype.

Principal component analysis was performed using the R princomp function. Of the measured variance in the transcriptome, 98.5% was captured in the first two principal components; therefore, the plot in Figure 2 only includes the first two components. To demonstrate the extent of the overlap, an ellipsoid hull for each class (BPD and control infants) was calculated as the ellipsoid of minimal area, such that all given points lie just inside or on the boundary of the ellipsoid [26]. An outline was then drawn of each corresponding ellipsoid in Figure 2. The two *P *values on a *t *test on the samples in each of the first two principal components were greater than 0.05, confirming the visual impression.

Hierarchical clustering (using the R hclust function) based on Euclidean distance of expression between samples was performed using all those genes of the 54,675 that had at least one value greater than the mean in the 54 samples.

## Abbreviations

BPD, bronchopulmonary dysplasia; ELGAN, Extremely Low Gestational Age Newborn; FDR, false discovery rate.

## Authors' contributions

JC directed the study, recruited participants, obtained samples, performed analyses, and wrote the manuscript. LJvM, provided guidance on study design and mentorship regarding clinical and epidemiologic aspects, and edited the manuscript. YS helped to conceive and obtain funding for the study. EA advised on the design and helped to conduct analyses in the study. AL advised on the design of the study, edited the manuscript, and helped to obtain additional phenotypic data. IK participated in the conception and direction of the study, completed bioinformatics analyses, and edited the manuscript.
